# Disability Identification and Self-Efficacy among College Students on the Autism Spectrum

**DOI:** 10.1155/2014/924182

**Published:** 2014-02-23

**Authors:** Paul T. Shattuck, Jessica Steinberg, Jennifer Yu, Xin Wei, Benjamin P. Cooper, Lynn Newman, Anne M. Roux

**Affiliations:** ^1^A. J. Drexel Autism Institute, Drexel University, 3020 Market Street, Suite 560, Philadelphia, PA 19104, USA; ^2^Washington University, One Brookings Drive, St. Louis, MO 63130, USA; ^3^SRI International, 333 Ravenswood Avenue, BS169, Menlo Park, CA 94025-3493, USA

## Abstract

The number of youth on the autism spectrum approaching young adulthood and attending college is growing. Very little is known about the subjective experience of these college students. Disability identification and self-efficacy are two subjective factors that are critical for the developmental and logistical tasks associated with emerging adulthood. This study uses data from the National Longitudinal Transition Study 2 to examine the prevalence and correlates of disability identification and self-efficacy among college students on the autism spectrum. Results indicate nearly one-third of these students do not report seeing themselves as disabled or having a special need. Black race was associated with lower likelihood of both disability identification and self-efficacy.

## 1. Introduction

Approximately 50,000 youth on the autism spectrum turn 18 years old each year, a proxy indicator for the number entering adulthood. About 35%, or 17,500 youth on the autism spectrum per year, go on to attend college within the first six years after high school [1]. Of the youth on the autism spectrum who attend college, about 34% choose a major in science, technology, engineering, or math (STEM). This rate of STEM majoring is significantly higher than that seen in any other disability category and also higher than the 23% rate seen in the general population [2, 3]. In the broader context of a competitive global economy, the subset of students who pursue STEM majors are especially important to consider.

Some research has documented how the core challenges of autism spectrum disorder (ASD)—difficulty maintaining the reciprocal interaction essential to learning, poor nonverbal communication, and a limited ability to understand and use the rules of social behavior—may limit success in college [4–6]. However, very few studies have examined subjective perceptions of self among postsecondary college students on the autism spectrum. This fact is at odds with the current federal priority to promote ASD research that emphasizes understanding the insights, personal experiences, and perspectives of people on the autism spectrum [7].

Two important components of self-perception that are central to the developmental challenges of transitioning from adolescence to emerging adulthood are identity formation and self-efficacy [8–10]. Identity refers to one's self-image and has multiple facets including racial and ethnic identity, gender identity, and disability identity. Identity formation is a dynamic, nuanced, multidimensional, and lifelong process that takes on particular importance during emerging adulthood when questions about life purpose and direction move to the foreground [11, 12]. The emergent sense of identity during this period has ramifications for decision making related to college majors, persistence to degree completion, career choices, and relationship formation.

Particularly salient for emerging adults on the autism spectrum is disability identity. The literature regarding disability and identity includes complex definitions and debates about the social versus medical model of disability and what it truly means to be “disabled” [13–15]. Clearly, identity formation is a highly nuanced topic. For the purposes of this exploratory paper, we rely on a self-report measure of seeing oneself as having a disability or special need. We argue that this singular dimension of disability identity is more appropriately referred to as “disability identification.” We were not able to find studies focusing on disability identity or identification exclusively among college students on the autism spectrum. We did find one report that described rates of disability identification among a sample of postsecondary youth (a mix of college students and nonstudents) with a variety of disability types [16]. Approximately three-quarters (76.1%) of youth on the autism spectrum in that study considered themselves individuals with a disability, but this rate was not broken out for college students.

Self-efficacy refers to one's perceived ability to do things for one's self and be successful [8]. A strong sense of self-efficacy has been recognized in developmental research as a component of resilience and an asset that helps foster healthy development [10]. Studies have shown a connection between a student's sense of self-efficacy and positive postsecondary education outcomes, such as higher grade-point averages and persistence [17–20], and specifically for completion rates and grades in STEM fields [21–23]. Although these studies draw a clear connection between self-efficacy and postsecondary success, there are very few studies that look at the issue for students on the autism spectrum.

Prior research has found that self-efficacy beliefs among STEM students with disabilities are malleable and have a reciprocally influential relationship with academic success and persistence [24]. Exploring the association between STEM majoring and self-efficacy is an important step toward a better understanding of which factors might promote success in a segment of the ASD population who could be especially valuable contributors to the national economy.

*Study Aims*. This exploratory study is one of the first of its kind to consider the subjective experiences of college students on the autism spectrum by examining disability identification and self-efficacy. In this study we use data from a large national survey to examine two questions. First, what are the distributions of disability identification and self-efficacy in college students on the autism spectrum? Second, what are the correlates of disability identification and self-efficacy in this group?

Given the lack of prior research on these topics among college students on the autism spectrum, we opted to examine a small set of correlates that were available in this secondary data set and are known to be significantly associated with a range of postsecondary outcomes for young adults on the autism spectrum: sex, race, household income, functional skills, and STEM major [1, 25–28]. Difficulty with social communication, another covariate in this study, is a defining feature of ASDs and is a consistently strong predictor of variability in adult outcomes [29].

## 2. Methods

### 2.1. Ethics Statement

Use of these data is governed by a data use agreement with the US Department of Education and was deemed exempt by the Drexel University Institutional Review Board.

### 2.2. Study Sample

Data for this study came from the National Longitudinal Transition Study 2 (NLTS2) conducted by SRI International under contract with the US Department of Education. The NLTS2 was conducted over 5 waves, each 2 years apart, from 2001 to 2009. At baseline, all youth were ages 13–16 years old and receiving special education services. The NLTS2 began with over 11,000 students, including 920 in the autism special education category.

The NLTS2 used a nationally representative two-stage sampling design. Local education agencies (LEAs) were sampled first followed by selection of students from special education enrollment rosters. Weighted estimates generalize to the national population of youth who had been receiving special education services at baseline [30]. Full details of the sampling strategy for NLTS2 were previously published [31]. The dependent variables for the current study are from the youth survey at wave 5, collected in 2009. This wave of NLTS2 had the largest number of postsecondary youth available for analysis. Unweighted sample numbers in this report were rounded to the nearest ten, as required by the US Department of Education.

For the sake of official special education enrollment reports, each student is counted only once in a primary disability category. Autism is one of twelve primary disabilities reporting categories mandated by the Individuals with Disabilities Education Act (IDEA) and included in the NLTS2. Each student's eligibility for special education services was determined by the school district from whose roster the student was sampled. Population-based research in the US has consistently found that the vast majority (>95%) of children receiving special education services in the autism category also meet *DSM-IV*-based case criteria for an ASD [32, 33].

There were approximately 660 participants identified in the autism category at the beginning of the NLTS2 who remained as participants at wave 5. Approximately 190 of these 660 youth had attended either a 2-year or 4-year college at some point since exiting high school. The total number of college attendees with valid values on all dependent measures was approximately 120—the total *N* for this study. Some only attended a 4-year college (13.9%). The rest either only attended a 2-year college (42.0%) or attended both 2- and 4-year colleges (44.1%) during the first eight years after high school. They ranged in age from 21 to 25 years old.

### 2.3. Dependent Measures

*Disability Identification*. The NLTS2 Youth Survey included a yes/no question about disability identification—“Some people have a disability or special need that makes it hard for them to do some things. Do you consider yourself to have any kind of disability or special need?”

*Self-Efficacy*. Valid self-efficacy questions should be phrased in ways that ask about a person's perceived capability—what they believe they can do or know how to do [34]. We carefully reviewed all NLTS2 Youth Survey questions to find items that were either worded very similarly to items from validated self-efficacy scales or had high face validity based on Bandura's guidelines for the construction of self-efficacy measures [34]. We found three questions asking youth how well each statement described him/herself: (1) “You can handle most things that come your way,” (2) “You know how to get information you need,” and (3) “You can get school staff and other adults to listen to you.” For each statement, the ordinal responses were (1) not at all like me, (2) a little like me, and (3) very much like me. Question 1 is nearly identical to an item from Schwarzer's widely used Generalized Self-Efficacy scale: “I can usually handle whatever comes my way” [35]. Asking about perceived ability to obtain needed information is commonly used in measures of self-efficacy among patients and students [34, 36]. Asking about perceived ability to be assertive and communicate effectively with teachers and adults is also common in measures of student self-efficacy [34].

### 2.4. Covariates

College major was split into STEM and not-STEM. STEM majors included computer science/information technology, engineering, mathematics, and other hard sciences such as biology, physics, and chemistry. Demographic covariates included sex, race, and household income. The income variable was collapsed into 4 ordinal categories for stratifying the descriptive point estimates and then rescaled to increments of $10,000 for the regression models to make odds ratios and coefficients interpretable as the estimated unit change in each dependent variable per $10,000 change in income. A 3-category, parent-report ordinal variable measured how well each youth can carry on a conversation: (1) “Does not carry on a conversation at all” or “Has a lot of trouble carrying conversation,” (2) “Has a little trouble carrying conversation,” and (3) “Has no trouble carrying conversation.” A functional skills scale was constructed by summing eight 4-category (not at all well, not very well, pretty well, and very well) parent-report questions about how well a youth could do the following tasks without help: tell time on an analog clock, read and understand common signs, count change, look up telephone numbers and use a telephone, get to places outside the home, use public transportation, buy own clothes at a store, and arrange a plane or train trip (Cronbach's alpha = 0.85 in the ASD group).

### 2.5. Data Analysis

Rates of missing data per covariate ranged from 0% to 15%, with two variables missing more than 10% (functional skills scale 11%; income 15%). Missing covariates were imputed using sequential regression in IVEware (version 0.1) to create 50 sets of data with no missing values [37, 38].

All reported estimates were weighted and variances adjusted to account for the complex sampling and the multiple imputations using the “mi svy” procedures available in Stata v12 which uses standard methods for combining estimates in the analysis of multiply imputed data [39]. Univariate point estimates and 95% confidence intervals were computed for describing the independent and dependent variables (Table 1). A logistic regression model estimated the adjusted association between correlates and the disability identification indicator (Table 2). Linear regression models estimated the adjusted associations with each ordinal self-efficacy item. We also estimated these models using an ordinal logistic method and found no differences in which covariates were found to be significantly associated with outcomes. We chose to report the linear regression findings for ease of interpretation.

Given the small sample size and the exploratory nature of our research aims we chose an alpha level of 0.10 for indicating statistical significance in tables. This choice decreases the likelihood of making a Type II Error (i.e., determining there is no effect in the population when there really is) while increasing the likelihood of making a Type I Error (i.e., determining there is an effect in the population when there really is not). Readers should interpret findings of statistical significance with caution.

## 3. Results

Reviewing the characteristics of the population of youth on the autism spectrum who attended either a 2-year or 4-year college or university since leaving high school (Table 1), these youth were more likely to be male (85.2%) and were concentrated in the higher income categories (38.7% with income $50,001–$75,000 and 37.3% with family income >$75,000).

Approximately two-thirds (69.4%) of youth on the autism spectrum considered themselves to have a disability or special need. Overall, college students on the autism spectrum reported a high level of confidence in their ability to “get information they needed” and “getting school staff and other adults to listen to them” (approximately 72% reported these statements described them “very much”), but lower confidence for “can handle most things that come their way” with only 40.9% reporting “very much.”

Regression model results (Table 2) revealed that the odds of disability identification were significantly lower among blacks and those with higher functional skills. Believing “one can handle most things that come their way” was significantly lower among STEM majors but higher among males and those with better conversation ability. Belief in being able to get needed information was lower in males but higher among those with better conversation ability. Belief in being able to get people to listen was lower among blacks (versus whites). Identity and self-efficacy measures were not significantly associated with each other in these models.

## 4. Discussion

Several important findings emerged from this study. Approximately one-third of college students on the autism spectrum do not consider themselves to have a disability or special need. Some colleges are now offering support programs targeted at students on the autism spectrum [40, 41]. If students are required to self-identify as being on the autism spectrum before qualifying for services then this may unintentionally exclude a large proportion of those in need. Colleges might consider options for reducing stigma by creating services to support social and academic success that are marketed to all students without requiring students to “out” themselves with respect to disability status. This is already done at many colleges for general academic tutoring and help with writing.

There can be both costs and benefits to accepting or rejecting disability identity. Lower rates of mental health problems have been associated with rejecting disability identity [42]. However, to succeed in college, many students on the autism spectrum will need supportive services and accommodations [43], and college students generally need to disclose and document their disability in order to receive accommodations and services through a college's disability services program. Strategically presenting one's disability is a key factor in obtaining disability services at postsecondary institutions [44, 45]. The transition planning process during high school needs to include a clear exploration of the potential pros and cons of disability disclosure on an individualized basis.

Less than half of college students on the autism spectrum expressed a high degree of global expectancy that they could “handle most things that come their way.” However, self-efficacy was higher on measures of getting information needs met and getting people to listen. Controlling for other factors, better conversation ability was significantly associated with higher self-efficacy on two measures: handling most things and getting needed information. Blacks had a lower likelihood of seeing themselves as disabled and a lower self-efficacy rating related to getting others to listen. This particular finding underscores the importance of examining racial disparities, a topic that has frequently been ignored by research on outcomes and services for adults on the autism spectrum [46]. Together, these findings suggest the need for interventions to improve self-efficacy and an understanding of disability identification, especially among blacks and those with more severe impairments.

Students with STEM majors reported lower self-efficacy on the measure of being able to “handle most things that come their way.” At first glance, this might seem to suggest STEM majors on the autism spectrum need to be targeted for supportive assistance. However, a recent study found that ASD students in STEM majors who only attended a 2-year college had a higher likelihood of persisting compared with their non-STEM ASD peers [2]. This counter-intuitive finding about self-efficacy and college persistence among STEM majors deserves further investigation. It could be that there are aspects of participating in a STEM major that buffer the impact of lower self-efficacy. It is also possible that STEM majoring presents students with greater challenges than non-STEM majoring, thereby lowering the sense of self-efficacy.

Future research should examine whether the students who do not self-report having a disability truly believe they have no disability or simply do not wish to acknowledge their disability in the context of a survey. Furthermore, there needs to be a careful examination of the link between disability identification and the formal disability disclosure and self-advocacy required to receive needed supports and accommodations during college.

We acknowledge several limitations of these findings. Self-efficacy has been measured many different ways in past research. As noted, we carefully selected three questions based on face validity and consistency with questions from validated instruments. However, we cannot tell with absolute certainty the degree to which they correspond with validated measures of self-efficacy. Our results cannot directly support a directional causal conclusion about the relationship between communication skills and self-efficacy. Additionally, disability identification was measured at one point in time. However, self-report of having a disability may not be temporally stable. The analysis plan was constrained by the list of covariates available in the data set—an inherent challenge when working with secondary data. Finally, the NLTS2 sampling approach made it impossible to examine the experiences of youth on the autism spectrum who did not participate in special education during high school compared to who did so but under a different reporting category than autism.

Strengths of this study include the nationally representative nature of the data, yielding population-level results reflective of the socioeconomic diversity of college students with ASD. A unique strength of this report is the use of self-report data to learn more about the subjective experience of this population.

## Figures and Tables

**Table 1:** Characteristics of college students on the autism spectrum.

Variable	Percentages (95% confidence interval)
Male	85.2 (65.0, 94.7)
Race	
White	83.3 (70.0, 91.5)
Black	8.8 (4.4, 16.6)
Other race	7.9 (2.4, 22.6)
Parent or guardian household income	
Up to $25,000	7.7 (3.7, 15.3)
$25,001–$50,000	16.3 (7.9, 30.2)
$50,001–$75,000	38.7 (25.7, 53.5)
More than $75,000	37.3 (22.2, 55.3)
How well youth converses	
No ability/lot of trouble	7.7 (4.0, 14.3)
Little trouble	66.2 (52.9, 77.3)
No trouble	26.2 (16.1, 39.5)
Had a STEM major	40.8 (25.8, 57.9)
Dependent measures	
Disability identification (youth considers self to have a disability or special need)	69.4 (57.6, 79.1)
Self-efficacy indicators	
(1) “You can handle most things that come your way”	
Not at all like me	4.3 (2.1, 8.5)
A little like me	54.8 (42.1, 66.9)
Very much like me	40.9 (29.8, 53.1)
(2) “You know how to get information you need”	
Not at all like me	2.7 (1.2, 6.1)
A little like me	25.2 (14.9, 39.3)
Very much like me	72.1 (58.4, 82.7)
(3) “You can get school staff and other adults to listen to you”	
Not at all like me	5.5 (2.8, 10.6)
A little like me	22.8 (14.4, 34.0)
Very much like me	71.7 (60.1, 81.0)

Source: National Longitudinal Transition Study 2.

Notes: number of multiply imputed data sets = 50. Weighted to population levels. Variances adjusted for sampling method.

**Table 2:** Regression model results.

Covariate	Disability identification (logistic regression odds ratios)	Self-efficacy (linear regression coefficients)		
		“You can handle most things that come your way”	“You know how to get information you need”	“You can get school staff and other adults to listen to you”
STEM major	0.7	−0.3*	0.1	0.0
Male	0.5	0.4^!^	−0.3*	−0.1
Race				
White	Reference	Reference	Reference	Reference
Black	0.1*	−0.1	−0.1	−0.5**
Other race	0.9	−0.2	−0.3	−0.1
Parent or guardian household income ($10 K increments)	1.0	0	0	0
Conversation ability	0.6	0.3*	0.3**	0.1
Functional skills scale	0.9^!^	0.0	0.0	0.0
“You can handle most things that come your way”	0.6			
“You know how to get information you need”	0.5			
“You can get school staff and other adults to listen to you”	1.9			
Disability identification		−0.1	−0.1	0.1

^!^ *P* < 0.10, *P < 0.05, **P < 0.01.

Source: National Longitudinal Transition Study 2.

Notes: number of multiply imputed data sets = 50. Weighted to population levels. Variances adjusted for sampling method.
